# Phytochemical profile influences the e-tongue responses, antioxidant, anti-inflammation, and hypoglycemic effects of wampee fruits from different regions

**DOI:** 10.1016/j.fochx.2025.102860

**Published:** 2025-08-05

**Authors:** Wen-hui Deng, Ying-ying Zheng, Yuan Li, Hong Zhang, Kai-xuan Lin, Han-Zhang Jiang, Li-Dan Fu, Hao-Dong Cui, Xin-yue Tong, Ziyang Hu, Nan Yang, Shi-yun Feng, Chi Shu, Han-Bin Lin

**Affiliations:** a Guangzhou University of Chinese Medicine, Guangzhou, Guangdong, China; b College of Food Science, Shenyang Agricultural University, Shenyang, Liaoning, China; c School of Pharmaceutical Sciences, Southern Medical University, Guangzhou, Guangdong, China; d Zhongshan Institute for Drug Discovery, Shanghai Institute of Materia Medica, Chinese Academy of Sciences, Zhongshan, Guangdong, China; e Department of Cardiology, Zhongshan Hospital of Traditional Chinese Medicine Affiliated to Guangzhou University of Traditional Chinese Medicine (Zhongshan Hospital of Traditional Chinese Medicine), Zhongshan, Guangdong, China; f State Key Laboratory of Chemical Biology, Shanghai, Institute of Materia Medica, Chinese Academy of Sciences, Shanghai, China; g University of Chinese Academy of Sciences, Beijing, China; h Perfect Life Science Research Institute (PLSRI), Perfect (GuangDong) Co., Ltd., Zhongshan 528402, China

**Keywords:** Anti-oxidant, Anti-inflammatory, Hypoglycemic, Metabolomics, Random forest tree analysis, Wampee

## Abstract

Wampee is a subtropical fruit valued for its health benefits. This study aimed to explore the relationship between its phytochemical composition, electronic tongue (e-tongue) responses (interred to be tastes), and bioactivities. The composition of Wampee extracts from different regions were subjected to detections of phytochemical composition, taste profiles, and common biological activities. 2568 metabolites were identified, primarily flavonoids, alkaloids, amino acids, and phenolic acids. The e-tongue system classified the samples into six clusters. Bioactivities varied among samples, with W6 (Yunan seedless) showing the strongest antioxidant potential, and W6 and W7 (Lipu chicken-heart) exhibiting the highest anti-inflammatory effects. Phenolic acids, flavonoids, and lipids contributed more to antioxidant capacity, while alkaloids and flavonoids played key roles in anti-inflammatory activity. This study highlights the phytochemical profiles of wampee fruits from different regions and shows a potential link between their metabolic features, tastes, and bioactivities.

## Highlights

• Phytochemical composition of nine wampee varieties were identified.
• Flavonoids are the main constituents of wampee from different regions.
• Wampee fruites from different regions show distinct tastes and bioactivities.
• Phenolic acids, flavonoids, alkaloids, and lipids are major bioactivity contributors.

## List of the chemical compounds studied

2’-Deoxycytidine

4-caffeoylshikimic acid

5’-Deoxyadenosine

5-hydroxyisovanillic acid

α-Ketoglutaric acid

Ala-Leu-Leu

Cinnamamide

Kaempferol-3-O-rutinoside

Vanilloloside

## 1 Introduction

*Clausena lansium* (Lour.) Skeels, commonly known as wampee, is a subtropical species of the Rutaceae family originating from China and has been widely cultivated in Southwest China for over 1500 years (Fan et al., 2018). Through selective breeding, more than 30 varieties of wampee have been developed worldwide, including in countries such as India, Vietnam, and Thailand (Wang, 2013). Currently, 11 varieties are cultivated in the Chinese provinces of Guangdong, Guangxi, Hainan, Fujian, and Taiwan (Luo, Chen, Zeng, Ruan, & Liu, 2023). The major macronutrient components of wampee pulp include protein, fat, carbohydrates, and dietary fiber, while the micronutrient content includes vitamin C, vitamin E, potassium, calcium, and others (Huang, Wang, Zhong, & Xu, 2023). However, the chemical composition of wampee can vary substantially depending on the cultivar, growing conditions, geographic factors, and even the analytical methods used (Li et al., 2005; Lim, 2012; Yu, Zhao, Gao, & Luo, 2022). In traditional Chinese medicine, wampee has long been used for medicinal purposes. For instance, the root, bark, stem, and leaves of wampee have been employed in the treatment of fever, cough, asthma, malaria, dermatological conditions, and rheumatism (Du et al., 2015). Fully ripened pulp can be used to manage digestive disorders, gastrointestinal diseases, and bronchitis (Chinese Flora, 1994). In our previous study, extracts of wampee fruit were shown to attenuate metabolic cardiomyopathy in mice by modulating the gastrointestinal microbiota toward a more balanced state (Shu, Xing, Zhao, et al., 2024). Consequently, in recent years, wampee has emerged as a focus of interest in both food processing and functional food research. In China, wampee cultivation has expanded significantly, generating substantial economic benefits for local communities. It is estimated that the production value associated with wampee may increase to tens of billions of Chinese yuan in the coming decades (Luo et al., 2023).

Currently, the nutritional application of wampee primarily relies on its edible portion, namely the pulp. The low fat and sugar contents in wampee result in a relatively low energy value of approximately 47 kcal per 100 g, while its high vitamin C (548 mg/kg) content makes it a valuable dietary supplement for essential vitamins and minerals (Wall, 2006a, Wall, 2006b). In addition to the aforementioned macro- and micronutrients, wampee is also rich in essential oils, phenolic compounds, and alkaloids. These bioactive compounds exhibit potent health-promoting and pharmacological properties, including anti-inflammatory, antibacterial, and anticancer activities, and they also contribute to the fruit's distinctive aroma and flavor (Huang et al., 2023; Wang et al., 2024). As these compounds play important roles in both the nutritional value and flavor of wampee, and ultimately influence its economic value, it is essential to conduct a comprehensive exploration of the fruit's chemical composition. However, most existing studies on the chemical composition of wampee have been limited to preliminary descriptive analyses and have failed to establish a clear connection between its chemical constituents, nutritional value, and flavor. In a study by Fan et al., the authors conducted a comprehensive analysis of the chemical composition of different parts of wampee using an untargeted UPLC-Q-Orbitrap-MS metabolomics strategy; however, their work primarily focused on metabolic pathways rather than linking chemical profiles to nutritional and sensory attributes (Fan et al., 2020). Similarly, Zhang et al. investigated tissue-specific specialized metabolism in different wampee varieties but did not connect their findings to the fruit's nutritional content or flavor characteristics (Zhang et al., 2024). Given that wampee is primarily consumed as a fresh fruit, limiting analysis to chemical composition alone reduces the practical value of the data. A thorough investigation of the relationship between chemical composition, nutritional value, and flavor will undoubtedly promote the broader application and cultivation of wampee.

In the current study, we aimed to conduct a comprehensive investigation into the potential role of the chemical composition of wampee in determining its electronic tongue (e-tongue) responses (inferred to represent taste) and biological activities. To this end, broad-target metabolomics analyses were performed on nine wampee samples from different regions, focusing on major compound classes. Subsequently, the effects on cell proliferation potential, anti-inflammatory, antioxidant, and hypoglycemic effects of the extracts from these samples were evaluated using a series of bioassays. The potential relationships between chemical composition and biological activities were then analyzed. Additionally, the e-tongue response profiles of the nine samples were examined in relation to their chemical composition. We hope that the findings from this study will provide valuable insights into the contributions of specific compounds to the biological effects and e-tongue response of wampee, and contribute to the establishment of standards for evaluating the value of wampee as food or medicinal resources.

## 2 Materials and methods

### 2.1 Sample preparation

**Fig. 1:**
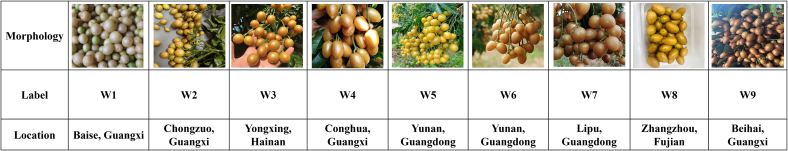
Representation images of morphology and location details of the nine wampee fruits collected in the current study. The commercial names of the fruits are represented by W1-W9 and the detailed information of the fruits is shown in Table S1.

Fruits of nine commercially available wampee were obtained from different regions of China, with detailed information regarding these samples provided in Fig. 1 and Table S1. Fresh samples with seeds removed were immediately sent for broad-target metabolomics analysis, while the remaining portions were stored at −80 °C for extraction. To prepare total composition extracts of wampee, the fruits (including peel and pulp) were dried and ground into 50-mesh powders. The full composition of the fruit powder was extracted using ultrasonic-assisted extraction, as previously described by Fan et al. (Fan et al., 2020). The resulting extracts were then analyzed for the e-tongue profiles and biological activities. The total sugar, total acid, and total phenol content (TPC) of the various cultivars were determined based on the method reported by Zhong et al. (Zhong et al., 2024).

### 2.2 Metabolomics detection and analyses

Samples were initially subjected to vacuum freeze-drying and then ground into a fine powder. Approximately 50 mg of the powdered sample was weighed and mixed with 1200 μL of pre-cooled 70 % methanol–water extraction solution. The mixture was vortexed six times at 30-min intervals and subsequently centrifuged at 12,000 rpm for 3 min. The resulting supernatant was filtered through a 0.22 μm microporous membrane and stored in injection vials for analysis. The chemical composition of the extracts was analyzed using ultra-performance liquid chromatography coupled with tandem mass spectrometry (UPLC-MS/MS). Chromatographic separation was performed on an Agilent SB-C18 column (1.8 μm, 2.1 mm × 100 mm) under gradient elution conditions, with mobile phases consisting of water containing 0.1 % formic acid (phase A) and acetonitrile containing 0.1 % formic acid (phase B). Mass spectrometry was carried out in multiple reaction monitoring (MRM) mode using an electrospray ionization (ESI) source. Key operational parameters included a source temperature of 500 °C and ion spray voltages of +5500 V and − 4500 V for positive and negative ion modes, respectively. Raw data were preprocessed for chromatographic peak extraction, integration, and quality control. Control samples were used to assess instrument reproducibility and ensure analytical consistency. Metabolites with a coefficient of variation greater than 0.5 were excluded to retain only those with robust repeatability. Statistical analyses included principal component analysis (PCA) to visualize clustering patterns among sample groups, and orthogonal partial least squares discriminant analysis (OPLS-DA) to identify significant metabolites. Differential metabolites were selected based on variable importance in projection (VIP) scores greater than 1 and fold-change thresholds of ≥2 or ≤ 0.5. Pathway enrichment analyses were conducted using the KEGG and MetMap databases to map identified metabolites to relevant biological pathways. Hypergeometric tests were employed to assess the statistical significance of pathway enrichment. Visualizations—including volcano plots, radar charts, and heatmaps—were generated to facilitate interpretation. Differential abundance scores were calculated to reflect pathway-wide metabolic trends. The results were evaluated and interpreted in the context of biological relevance, supporting the identification of key metabolic alterations and their underlying mechanisms.

### 2.3 Detection of e-tongue responses

The e-tongue response of wampee was evaluated using an SA402B electronic tongue system (Insent, Japan), equipped with five taste sensors and two reference sensors, following the manufacturer's instructions. For sample preparation, 5 g of each wampee fruit sample was mixed with 50 g of deionized water, thoroughly stirred, and then centrifuged at 4000 rpm for 15 min. A 30 mL aliquot of the filtered supernatant was used for electronic tongue analysis. The sample was introduced into the sensor probes of the SA402B system, and measurements were recorded continuously over a 30-s period. To minimize cross-sample contamination, the sensors were rinsed with a reference solution for 120 s between measurements. All analyses were performed at a constant temperature of 25 ± 1 °C. The four most consistent datasets were selected for further analysis. Sensor data were used to determine the relative intensities of different taste profiles, including umami, salty, sour, astringent, and bitter tastes. The five taste sensors corresponded to specific taste categories: AAE for umami (savory), CTO for salty, CA0 for sour, AE1 for astringency, and C00 for bitterness. The two reference sensors were used for calibration and baseline correction.

### 2.4 Detection of cell proliferation using methylthiazolyldiphenyl-tetrazolium bromide (MTT) assay

The effects of different wampee extracts on the viability of Caco-2 cells (4 × 10^4^/mL) were assessed to evaluate the effects of wampee extracts on cell proliferation potential using MTT assays (Ng, Lam, & Fong, 2003). Briefly, cells were incubated with different extracts (2 mM) for 24 h, after which the culture supernatant was discarded. The cells were then incubated with 10 μL of MTT solution at 37 °C for 1 h. The optical density (OD) at 450 nm was measured using a microplate reader (ELX-800, BIOTEK, USA) and used as an indicator of cell viability.

### 2.5 Detection of changes in body weight and blood parameters in SD rats after fed with different wampee samples

For *in vivo* assessment, Sprague-Dawley (SD) rats (eight weeks old, weighing approximately 250 g) were purchased from Changsheng Biotechnology (Liaoning, China). The rats were fed diets containing different wampee extracts (50 mg/kg body weight) daily for two weeks, and changes in body weight were monitored (Shu et al., 2024). After the two-week feeding period, the rats were euthanized using an overdose of pentobarbital sodium (250 mg/kg body weight), and blood samples were collected for subsequent analysis of blood parameters, including serum glucose, triglyceride, total cholesterol, low density lipoprotein cholesterin (LDL-C), and high density lipoprotein cholesterin (HDL—C). All animal experiments were conducted in accordance with the Guidelines for the Care and Use of Laboratory Animals (National Institutes of Health, 8th edition, 2011) and were approved by the Ethics Committee of Shenyang Agricultural University (approval No. 24022801, approved on February 28, 2024).

### 2.6 Detection of anti-oxidant effects of different wampee samples

#### 2.6.1 Cellular antioxidant activity (CAA) assay

For CAA detection, cells were suspended in serum-free medium at a density of 4 × 10^4^ cells/mL, and 100 μL of the suspension was added to each well of a 96-well plate, with three replicates per condition. After incubation at 37 °C in a 5 % CO₂ atmosphere for 24 h, the medium was removed and the cells were washed with PBS. Each well was then treated with 100 μL of serum-free medium containing 25 μmol/L DCFH-DA and the test sample at a concentration of 2 mg/mL, followed by a 60-min incubation at 37 °C in a 5 % CO₂ atmosphere. Subsequently, the medium was removed, and the cells were washed once with PBS before adding 100 μL of 600 μmol/L ABAP solution to each well. Fluorescence was measured using a microplate reader at an excitation wavelength of 485 nm and an emission wavelength of 538 nm every 5 min for a total duration of 60 min. Fluorescence intensity was plotted over time, and the area under the curve (AUC) was calculated to quantify antioxidant activity. Results were expressed as CAA values, with higher ABAP-induced fluorescence indicating stronger antioxidant effects.

#### 2.6.2 DPPH radical scavenging assay

For the DPPH radical-scavenging assay, a 75 mM phosphate-buffered saline (PBS) solution was prepared and adjusted to pH 7.4. DPPH was dissolved in ethanol to a final concentration of 0.2 mM. To assess antioxidant activity, 100 μL of each test sample (diluted 50-fold in PBS) was added to 100 μL of the 0.2 mM DPPH solution in a 96-well plate. Sample blanks (test sample with ethanol) and negative controls (DPPH solution with ethanol) were included. The plate was mixed thoroughly and incubated in the dark at room temperature for 30 min. Absorbance was measured at 517 nm using a microplate reader. DPPH radical-scavenging activity (%) was calculated using the formula: scavenging activity (%) = (A_0_ − A_1_) / A_0_ × 100, where A_0_ represents the absorbance of the negative control, and A1 represents the absorbance of the sample.

#### 2.6.3 ABTS radical cation scavenging assay

For the ABTS radical cation scavenging assay, a 7 mM ABTS solution was prepared and mixed with 2.45 mM potassium persulfate in a 1:1 ratio. The mixture was incubated in the dark at room temperature for 12–16 h to generate ABTS^+^ radicals. The resulting ABTS^+^ solution was then diluted with ethanol to achieve an absorbance of 0.70 ± 0.02 at 734 nm. To assess antioxidant activity, 10 μL of the diluted test sample was added to 200 μL of the ABTS^+^ solution in a 96-well plate. The plate was incubated at room temperature for six minutes, and absorbance was measured at 734 nm using a microplate reader. ABTS radical scavenging activity (%) was calculated using the formula: scavenging activity (%) = (A0 − A1) / A0 × 100, where A_0_ represents the absorbance of the ABTS^+^ solution, and A_1_ represents the absorbance of the test sample.

### 2.7 Detection of anti-inflammation effects of different wampee samples

The production of pro-inflammatory cytokines, including IL-6, IL-1β, and TNF-α, in RAW 264.7 cells treated with LPS and different extracts was evaluated using ELISA with specific kits (MEXN-M0042 for IL-6, MEXN-M0035 for IL-1β, MEXN-M0047 for TNF-α; Shanghai Meixuan Biological Science and Technology Ltd., Shanghai, China), according to the manufacturer's instructions. Briefly, cell samples were lysed with RIPA lysis buffer, and 10 μL of each sample was incubated with HRP-labeled detection reagents for 60 min at room temperature. Subsequently, 50 μL of substrate solutions A and B was added to each well and incubated for an additional 15 min at room temperature. The concentrations of the cytokines were determined by measuring the optical density (OD) at 450 nm using a MultiSkan3 microplate reader (170–3930, Thermo, USA).

### 2.8 Detection of hypoglycemic effects of different wampee samples

The α-amylase inhibitory activity of wampee extracts was assessed using a standard α-amylase enzyme inhibition assay. Briefly, the reaction mixture contained 250 μL of each extract and 200 μL of pancreatic α-amylase solution in a test tube. The reaction was initiated by adding 200 μL of a soluble starch solution and incubated at 37 °C for 30 min. The reaction was then terminated by adding 300 μL of DNSA (3,5-dinitrosalicylic acid) reagent. The mixture was further incubated at 100 °C for 20 min, after which the absorbance was measured at 540 nm using a microplate reader. The α-glucosidase inhibitory activity was evaluated by adding the extracts to 100 μL of 2 U/mL α-glucosidase derived from yeast. After adding 50 μL of potassium phosphate buffer (0.1 M, pH 6.8) to the enzyme/extract mixture, the solution was incubated at 37 °C for 12 min. The enzymatic reaction was then initiated by adding 35 μL of 0.005 M *p*-nitrophenyl-α-D-glucopyranoside (pNPG), and absorbance was monitored at 405 nm for one minute. A control experiment without test samples was included for comparison.

### 2.9 Statistical analyses

Data are presented as mean ± standard deviation (SD). One-way analysis of variance (ANOVA), followed by post-hoc comparisons using the Tukey method, was performed for normally distributed data. Metabolites with potential to distinguish e-tongue response profiles or specific biological activities among wampee cultivars were identified using random forest tree analysis (RFTA), implemented with the randomForest package in R (version 4.2.2). Spearman correlation analysis was used to assess the relationships among metabolites across different wampee samples. Statistical significance was considered when the two-tailed *P* value was less than 0.05.

## 3 Results

### 3.1 Characterization of different wampee samples

Nine wampee samples (W1–W9) were selected for subsequent assays based on their commercial availability. These wampee plants are mainly distributed in Southwest China, including the provinces of Guangdong, Guangxi, Fujian, and Hainan, and were sampled between May and June 2024 with similar temperature range and climates. Fruit sizes ranged from 1 cm (W1) to 5 cm (W4, W9), and detailed sampling locations and morphological characteristics of the samples are provided in Fig. 1 and Table S1. Additionally, total sugar, total acid, and total phenol content (TPC) of the different wampee samples were measured and are shown in Fig. S1: no significant differences were detected among the samples. Based on the detection results of total sugar, total acid, and TPC, there was no difference regarding the maturity between different wampee fruits.

### 3.2 *E*-tongue responses of different wampee extracts

**Fig. 2:**
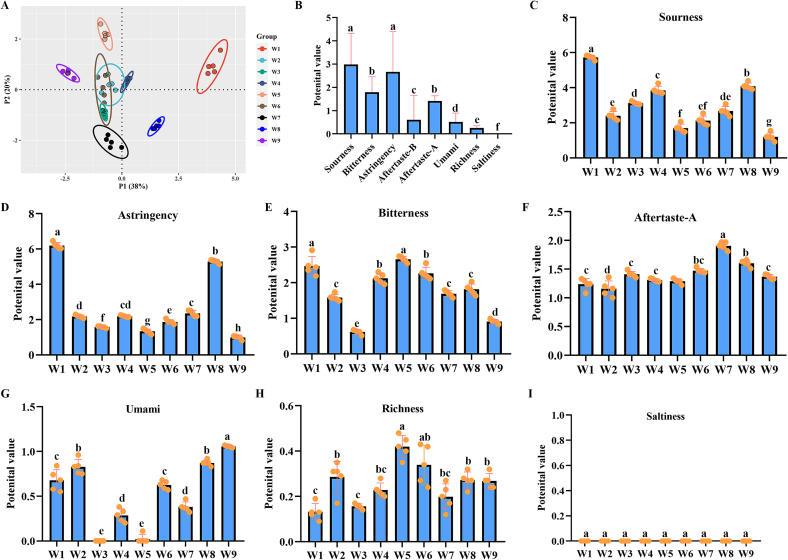
*E*-tongue response features of the nine wampee samples detected by an electronic tongue system. Eight parameters regarding e-tongue responses were detected, including sourness, bitterness, astringent, aftertaste-A, aftertaste-B, umami, saltness, and richness were detected using a SA402B electronic tongue system (*n* = 5). **A**, PCA analysis of the e-tongue response features of the nine samples. **B**, analysis results of the average value of different e-tongue responses of the nine samples. **C**, analysis results of the average value of sourness of the nine samples. **D**, analysis results of the average value of astringency of the nine samples. **E**, analysis results of the average value of bitterness of the nine samples. **F**, analysis results of the average value of aftertaste-A of the nine samples. **G**, analysis results of the average value of umami of the nine samples. **H**, analysis results of the average value of richness of the nine samples.

The e-tongue responses of different wampee fruits were evaluated using an SA402B electronic tongue system. A total of eight taste-related parameters were measured: sourness, bitterness, astringency, aftertaste-A, aftertaste-B, umami, saltiness, and richness. Based on principal component analysis (PCA) of the electronic tongue data, the nine extracts were classified into six distinct clusters (Fig. 2A), indicating that e-tongue response characteristics varied by geographic origin. Among the clusters, W2, W3, W4, and W6 exhibited similar overall e-tongue response profiles (Fig. 2A), while the remaining five extracts displayed more distinct e-tongue response characteristics. Detailed analysis revealed that the dominant e-tongue response attributes of wampee fruits were sourness and astringency (Fig. 2B), with W1 showing the highest levels of both (Fig. 2C and D). No saltiness was detected in any of the extracts (Fig. 2B). In addition, bitterness and aftertaste-A contributed to the overall e-tongue response profile of several extracts (Fig. 2E and F). Aftertaste-B was detected only in W3 and W4. Umami was observed in W1, W3, W4, W5, and W8 (Fig. 2G), while all extracts showed only weak richness, as indicated by the detection results (Fig. 2H).

### 3.3 Effects on cell proliferation and rat health of different wampee extracts

**Fig. 3:**
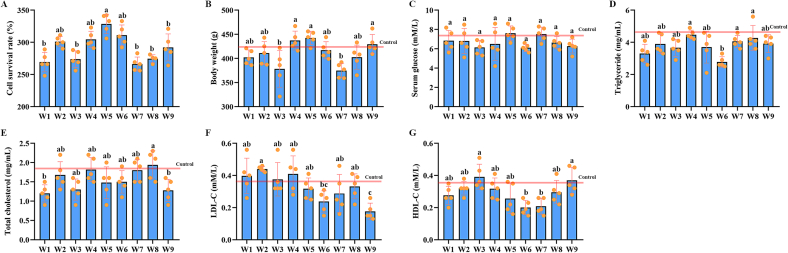
Potential influences of different wampee extracts on cell proliferation and health of SD rats. Caco-2 cells (4 × 10^4^/ml) were incubated with different extracts (2 mM) for 24 h and cell viability was determined using MTT assays. SD rats were then fed with diets containing different wampee extracts (50 mg/kg body weight) daily for two weeks, and changes in body weight, and blood parameters were then detected (n = 5). **A**, analysis results of the cell viability. **B**, analysis results of the body weight. **C**, analysis results of the blood glucose level. **D**, analysis results of the serum triglyceride level. **E**, analysis results of the serum cholesterol level. **F**, analysis results of the serum LDL-C level. **G**, analysis results of the serum HDL-C level.

The effects on cell proliferation potential of extracts from different wampee fruits was assessed using the MTT assay. As shown in Fig. 3A, no significant difference regarding effects on cell proliferation were observed for among extracts. Notably, extracts from W5 and W6 exhibited the strongest promotive effects on cell proliferation, but the difference compared was not statistically significant. Subsequently, SD rats were fed diets containing different wampee extracts for two weeks. The results showed regarding the effects on body weight, blood glucose, triglyceride levels, total cholesterol, LDL-C, or HDL-C (Figs. 3B–3G), only mild difference was detected with some extracts while the difference to control levels were all insignificant, confirming the safety and edible nature of wampee.

### 3.4 Antioxidant capabilities of different wampee extracts

**Fig. 4:**
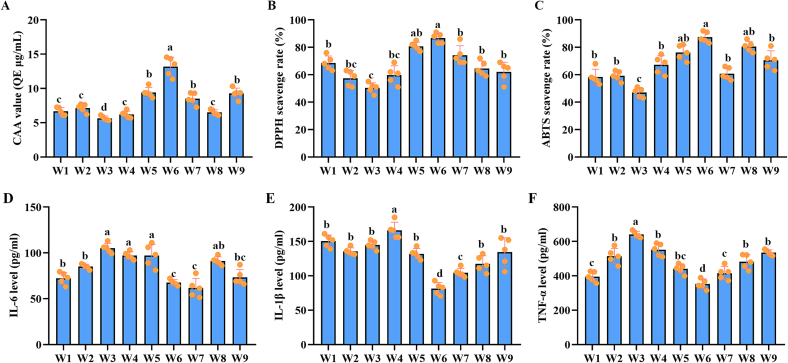
Potential anti-oxidant capabilities of different wampee extracts as detected by CAA, DPPH, and ABTS assays, respectively, and potential anti-inflammatory capabilities of different wampee extracts by ELISA detection of IL-6, IL-1β, and TNF-α (n = 5). **A**, analysis results of anti-oxidant capabilities of different wampee extracts detected by CAA assays. **B**, analysis results of anti-oxidant capabilities of different wampee extracts detected by DPPH assays. **C**, analysis results of anti-oxidant capabilities of different wampee extracts detected by ABTS assays. Different labels represents statistical difference between each two groups. **D**, analysis results of ELISA detection of IL-6. **E**, analysis results of ELISA detection of IL-1β. **F**, analysis results of ELISA detection of TNF-α. Different labels represents statistical difference between each two groups.

Extracts from different wampee fruits were subjected to CAA, DPPH, and ABTS assays to evaluate their antioxidant capacities. The CAA results indicated that extracts from W6 exhibited the strongest antioxidant activity, while extracts from W3 showed the weakest (Fig. 4A). Consistent findings were observed in the DPPH and ABTS assays, where the highest radical scavenging activities were again detected in W6 extracts, and the lowest in W3 extracts (Fig. 4B and C). However, the intermediate antioxidant capacities of the other extracts varied across the three assays (Fig. 4), which may be attributed to differences in detection principles and sensitivities among the assay methods.

### 3.5 Anti-inflammatory capabilities of different wampee extracts

The potential anti-inflammatory effects of extracts from different wampee fruits were assessed by incubating RAW 264.7 cells with LPS and the respective extracts. Compared with their antioxidant capacities, the anti-inflammatory activities of the extracts showed greater variation among the different cultivars. In terms of inhibiting IL-6 production, W7 demonstrated the strongest effect (Fig. 4D). In contrast, extracts from W6 showed the most potent inhibitory effects on IL-1β and TNF-α production (Fig. 4E and F). These differences may be attributed to the distinct compound compositions present in each extract.

### 3.6 Hypoglycemic effects of different wampee extracts

**Fig. 5:**
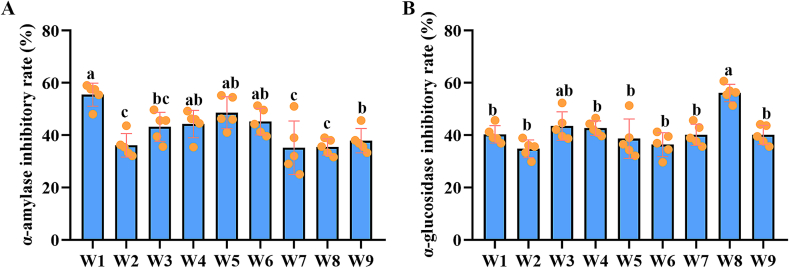
Potential Hypoglycemic effects of different wampee extracts by assessing inhibitory effects on α-amylase and α-glucosidase (n = 5). **A**, analysis results of inhibitory effects of different wampee extracts on α-amylase. **B**, analysis results of inhibitory effects of different wampee extracts on α-glucosidase.

The inhibitory effects on α-amylase and α-glucosidase are commonly used as indicators of the hypoglycemic potential of compounds. In the present study, extracts from W1 to W9 were tested for their inhibitory effects on these two enzymes. The results showed that extracts from W1 exhibited the strongest α-amylase inhibitory activity (Fig. 5A), while extracts from W8 demonstrated the most potent inhibition of α-glucosidase (Fig. 5B). Although the inhibitory effects of extracts from different wampee fruits varied at intermediate levels, the differences between groups were relatively small, suggesting that there were no substantial discrepancies in hypoglycemic potential among the different wampee extracts.

### 3.7 Metabolites identification and pathway enrichment

The chemical composition of different wampee fruits was determined using UPLC-MS/MS. The major classes of compounds detected included amino acids and derivatives, phenolic acids, nucleotides and derivatives, flavonoids, quinones, lignins and coumarins, tannins, alkaloids, organic acids, terpenoids, lipids, and others. Quality control (QC) analysis confirmed that the sample extraction and detection processes were stable and reproducible. In total, 2568 compounds were identified, including 1573 in positive ion mode and 995 in negative ion mode. Detailed information such as retention time, exact mass, molecular formula, compound type, match percentage, and CAS number is provided in Table S2.

**Fig. 6:**
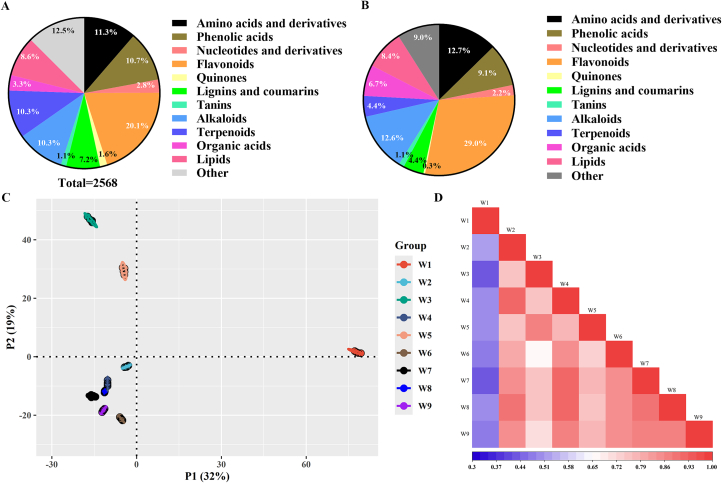
Phytochemical composition of different wampee fruits as detected by metabolomics detection (n = 5). **A**, phytochemical composition features of wampee samples by number of different chemical classes. **B**, phytochemical composition features of wampee samples by abundance of different chemical classes. **C**, PCA analysis of phytochemical composition features of wampee samples. **D**, correlation between different wampee samples regarding phytochemical composition features.

Across all nine samples, the identified compounds included 291 amino acids and derivatives, 276 phenolic acids, 73 nucleotides and derivatives, 517 flavonoids, 40 quinones, 186 lignins and coumarins, 29 tannins, 264 alkaloids, 264 terpenoids, 85 organic acids, 222 lipids, and 321 other compounds (Table S2; Fig. 6A). Flavonoids were the predominant constituents in wampee fruits, followed by amino acids and derivatives, phenolic acids, alkaloids, terpenoids, and lipids (Fig. 6A). In terms of relative abundance, flavonoids accounted for the highest proportion (29.0 %), followed by amino acids and derivatives (12.7 %) and alkaloids (12.6 %) (Fig. 6B).

Principal component analysis (PCA) revealed substantial variation in compound composition among the different wampee samples (Fig. 6C). W2, W4, W6, W8, and W9 exhibited relatively similar compositions, while W1, W3, and W5 showed distinct profiles compared to the others (Fig. 6C). Correlation analysis further indicated that W2 shared higher compositional similarity with W4 and W8; W3 was more closely related to W5; and W4 showed strong correlations with W2, W7, and W8. In contrast, W1 displayed low correlation with all other wampee samples (Fig. 6D).

Metabolites in plants are synthesized through complex metabolic pathways, regulated by various genes and influenced by environmental factors. In the present study, samples were collected from regions with similar climates and within the same time frame, ensuring that the detected metabolites were primarily produced under genetic regulation specific to each wampee samples. Pathway enrichment analysis was conducted using the KEGG database, and pairwise comparisons between groups were performed and presented in Fig. S2. However, the differences in KEGG pathway enrichment were complex due to the multiple comparisons among different wampee plants, and yielded limited information relevant to the processing or commercial value of wampee fruits.

### 3.8 Association between metabolites and features of different wampee extracts

**Fig. 7:**
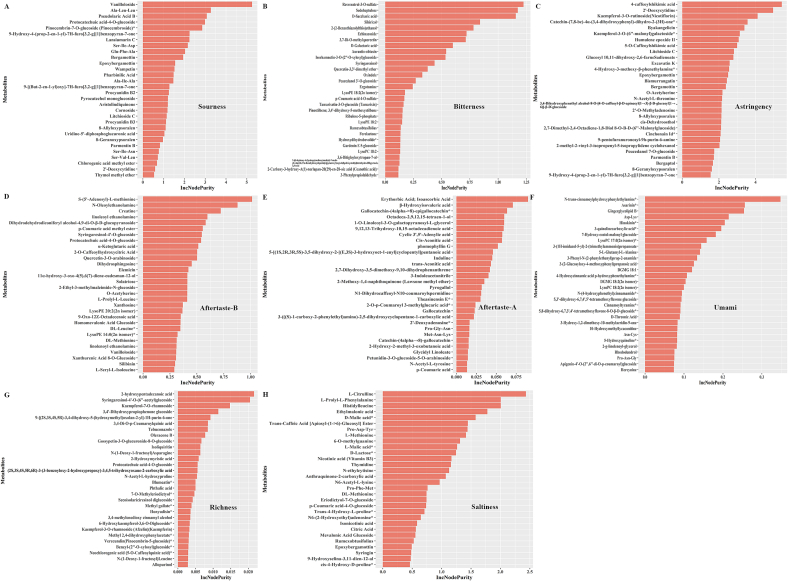
Key compounds influence the wampee e-tongue response analyzed by random forest tree analysis (RFTA). **A**, RFTA of key compounds influencing sourness in wampee samples. **B**, RFTA of key compounds influencing bitterness in wampee samples. **C**, RFTA of key compounds influencing astringency in wampee samples. **D**, RFTA of key compounds influencing aftertaste-A in wampee samples. **E**, RFTA of key compounds influencing aftertaste-B in wampee samples. **F**, RFTA of key compounds influencing umami in wampee samples. **G**, RFTA of key compounds influencing richness in wampee samples. **G**, RFTA of key compounds influencing saltiness in wampee samples.

**Fig. 8:**
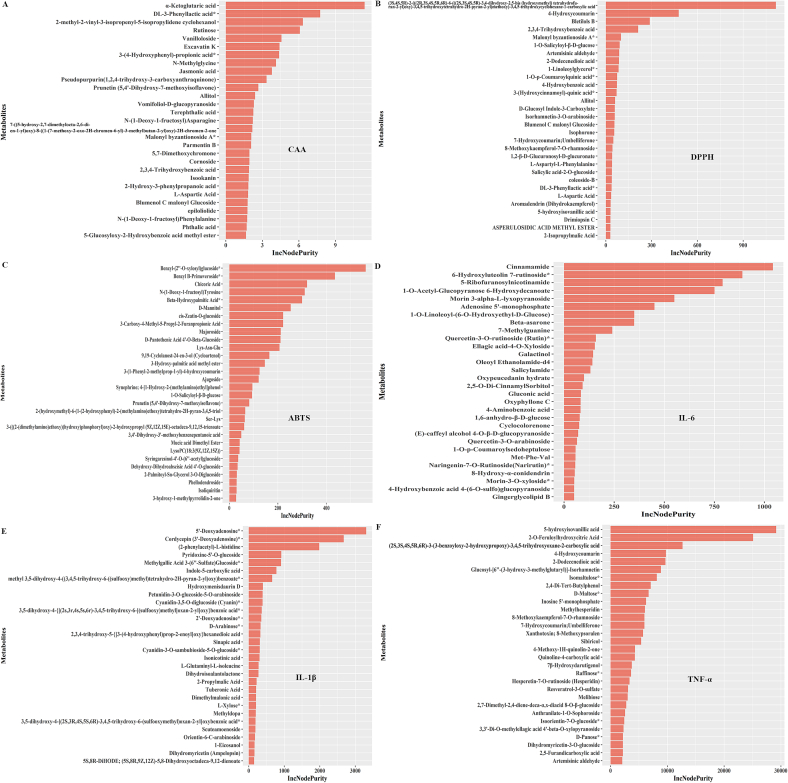
Key compounds influence different biological capacities in wampee samples. **A**, RFTA of key compounds influencing anti-oxidant capacity detected by CAA assays in wampee samples. **B**, RFTA of key compounds influencing anti-oxidant capacity detected by DPPH assays in wampee samples. **C**, RFTA of key compounds influencing anti-oxidant capacity detected by ABTS assays in wampee samples. **D**, RFTA of key compounds influencing suppressing effects on IL-6 in wampee samples. **E**, RFTA of key compounds influencing suppressing effects on IL-1β in wampee samples. **F**, RFTA of key compounds influencing suppressing effects on TNF-α in wampee samples.

As shown in Table S2, W1 exhibited the highest levels of alkaloids, amino acids and derivatives, lipids, nucleotides and derivatives, organic acids, and tannins. W9 had the highest level of flavonoids, W3 had the highest level of lignans and coumarins, and W6 showed the highest level of phenolic acids. No significant differences in the abundance of quinones and terpenoids were detected among the different wampee extracts. Although W1 had the most diverse and abundant metabolite composition, it did not exhibit superior biological activities compared to the other extracts, as previously described. Therefore, random forest tree analysis (RFTA) was performed to provide a preliminary explanation of the metabolite composition and distinguishing features of the different wampee extracts. As shown in Fig. 7, Fig. 8, the contributions of specific compounds to various traits were determined based on their IncNodePurity values.

In Figs. 7A–7H, the top 30 compounds with the strongest influence on different e-tongue response attributes are listed. Among the various e-tongue response profiles, sourness and astringency were primarily influenced by metabolite composition, while the other e-tongue responses showed minimal correlation with wampee composition based on IncNodePurity values. For sourness, vanilloloside and Ala-Leu-Leu demonstrated markedly stronger contributions than other compounds (Fig. 7A). Among the top 30 metabolites ranked by IncNodePurity, seven were amino acids and derivatives, and eight were lignans and coumarins, suggesting that these two compound classes may play a key role in determining the sour taste of wampee. In the case of astringency, 4-caffeoylshikimic acid had the highest contribution, followed by 2′-deoxycytidine, kaempferol-3-O-rutinoside, and catechin-(7,8-bc)-4α-(3,4-dihydroxyphenyl)-dihydro-2(3H)-one* (Fig. 7C). Among the top 30 contributing metabolites, four were flavonoids and 11 were lignans and coumarins, indicating that these two classes are likely central to the perception of astringency in wampee fruits. Based on the RFTA results, it appears that the e-tongue response profiles of wampee are partially determined by metabolite composition and may differ significantly across wampee plants.

Additionally, the influence of metabolite composition on the biological activities of wampee was analyzed as well. The data showed that, compared with e-tongue response attributes, the biological activities of wampee were more strongly influenced by metabolite composition, as indicated by higher IncNodePurity values (Fig. 8). However, due to variations in detection methods and indicator sensitivity, the major metabolites contributing to different biological activities varied considerably. For example, antioxidant capacity was assessed using a combination of CAA, DPPH, and ABTS assays. In the CAA assay, α-ketoglutaric acid was identified as a major contributor to antioxidant activity (Fig. 8A). Overall, the differences in contribution among metabolites in the CAA assay were relatively small. Among the top 30 metabolites ranked by IncNodePurity values, four were amino acids and derivatives, and eight were phenolic acids, suggesting that these two classes may play a primary role in determining the antioxidant potential of wampee as measured by the CAA assay.

However, in terms of antioxidant capabilities detected by the DPPH and ABTS detections, the contribution by different metabolites varied substantially. In DPPH detection, (3*S*,4*S*,5*R*)-2-(((2*R*,3*S*,4*S*,5*R*,6*R*)-6-(((2*S*,3*S*,4*S*,5*R*)-3,4-dihydroxy-2,5-bis (hydroxymethyl) tetrahydrofuran-2-yl)oxy)-3,4,5-trihydroxytetrahydro-2H-pyran-2-yl)methoxy)-3,4,5-trihydroxycyclohexane-1-carboxylic acid showed significantly stronger antioxidant capabilities than other metabolites (Fig. 8B). Among the top 30 metabolites ranked by IncNodePurity values, there were seven flavonoids, four lipids, six phenolic acids, and six terpenoids. In the ABTS assay, benzyl-(2”-O-xylosyl)glucoside exhibited the highest antioxidant contribution (Fig. 8C). Among the top 30 metabolites, there were four alkaloids, three flavonoids, and five lipids. Overall, these findings suggest that flavonoids, lipids, and phenolic acids play key roles in determining the antioxidant capacities of wampee fruits, particularly as assessed by DPPH and ABTS assays.

Similar analysis results were observed for the anti-inflammatory activities among different wampee extracts. Cinnamamide contributed most significantly to the suppression of IL-6 (Fig. 8D), while 5′-deoxyadenosine showed the strongest contribution to the inhibition of IL-1β (Fig. 8E), and 5-hydroxyisovanillic acid was the most influential in reducing TNF-α levels (Fig. 8F). Based on the composition of the top 30 metabolites identified in each assay, alkaloids and flavonoids appear to form the primary basis for the anti-inflammatory effects observed across different wampee extracts.

Collectively, the antioxidant and anti-inflammatory activities of wampee fruits varied and were substantially influenced by their metabolic compositions. However, identifying the key compounds responsible for these differences remains challenging and warrants further investigation in future studies.

## 4 Discussion

Wampee is a tropical fruit widely distributed in Southwest China, traditionally known for its therapeutic effects in alleviating fever, cough, asthma, malaria, dermatological conditions, and rheumatism (Du et al., 2015). In recent years, the cultivation of wampee has expanded significantly, contributing substantial economic benefits to local communities in China. However, research on the composition, nutritional value, and genetic characteristics of wampee has not kept pace with its agricultural development. Although some studies have explored various aspects of wampee, systematic and comprehensive evaluations remain limited. In the present study, we collected nine commercially available wampee samples from different regions in China and conducted assessments regarding their taste profiles (e-tongue responses), antioxidant, anti-inflammatory, and hypoglycemic properties. Additionally, the metabolic composition of each extracts was determined using broad-target metabolomics and analyzed in relation to the observed functional and e-tongue responses s. The results demonstrated that wampee fruits from different regions can be differentiated based on their e-tongue response characteristics and metabolic profiles. Furthermore, antioxidant and anti-inflammatory activities varied significantly among wampee extracts, with certain key metabolites likely contributing more prominently to specific biological effects.

The apparent taste profile of wampee as a fresh fruit typically includes sweetness, sourness, and astringency (Luo et al., 2023). In the present study, the SA402B electronic tongue system was used to evaluate eight taste parameters: sourness, bitterness, astringency, aftertaste-A, aftertaste-B, umami, saltiness, and richness. The results confirmed that sourness and astringency were the dominant e-tongue response attributes across the nine wampee samples. However, these findings were inconsistent with sensory perceptions reported by volunteers, who tended to prioritize sweetness as a favorable taste. Among the nine wampee samples, W1 exhibited the highest levels of both sourness and astringency, representing what volunteers identified as “the least favorable” flavor profile. Notably, W1 is primarily harvested from wild populations and is the smallest in size compared to the other samples. In addition to its strong sour and astringent e-tongue responses W1 showed a distinct overall e-tongue response composition, suggesting that artificial domestication may significantly improve the e-tongue response characteristics of wampee. Although wampee breeding is still in its early stages and remains unsystematic, these results indicate that targeted selection and cultivation could enhance sensory quality in future cultivars (Fan et al., 2021; Lu YuSheng et al., 2017; Wang et al., 2021).

Wampee has long been used in traditional Chinese folk medicine to alleviate a range of ailments, including fever, cough, asthma, malaria, dermatological conditions, and rheumatism (Chang et al., 2018; Huang et al., 2023). Despite the broad spectrum of biological activities reported for wampee fruits, only a few studies have explored their potential as functional foods or in adjuvant therapies. In our previous research, we investigated the effects of extracts from the W3 on metabolic cardiomyopathy (CMP), and the results demonstrated that the extract could ameliorate CMP-related symptoms by modulating gastrointestinal microbiota homeostasis (Shu et al., 2024). Supporting this, a recent study by Yang and Zhong (2025) reported that stachydrine, a known compound found in wampee, attenuates metabolic cardiac disorders associated with type 2 diabetes by restoring gut microbiota balance (Yang & Zhong, 2025). Beyond its cardiovascular benefits, another study by Song et al. revealed that a balasubramide derivative isolated from the W1 could alleviate acute lung injury by targeting the VDAC1 signaling pathway (Song et al., 2024). These studies highlight the novel biological potential of wampee, supporting the rationale for a systematic comparison of the biological activities among different wampee plants. In the present study, we evaluated the antioxidant, anti-inflammatory, and hypoglycemic effects of nine commercially available wampee fruites. Among them, W6 exhibited the strongest antioxidant capacity across multiple detection methods, while W5 and W8 also demonstrated notable antioxidant effects in specific assays. In terms of anti-inflammatory activity, all extracts showed considerable efficacy, with W6 and W7 exerting stronger inhibitory effects on pro-inflammatory cytokines compared to the others. These findings are consistent with previous reports on the antioxidant and anti-inflammatory properties of wampee (Huang et al., 2023; Wu et al., 2013; Zeng et al., 2024), further reinforcing the potential of wampee as a bioactive food source. Moreover, different wampee samples exhibited varying degrees of activity across the different biological assays, which may be attributed to differences in their metabolic compositions. In the current study, no significant hypoglycemic effects were observed in SD rats for any of the wampee extract. However, inhibitory effects on α-amylase and α-glucosidase were detected, suggesting potential for glycemic regulation. The absence of a measurable hypoglycemic effect *in vivo* may be related to the dosage or frequency of extract administration. These findings highlight the need for further investigation into the hypoglycemic potential of wampee, including optimal dosage, delivery strategies, and identification of active components. Collectively, given its promising ability to alleviate symptoms related to various disorders—particularly within the respiratory and digestive systems (Huang et al., 2023; Musa et al., 2022; Rodrigues, de Brito, & de Oliveira Silva, 2018). Future studies should aim to elucidate the molecular mechanisms underlying its biological functions and explore novel applications in both nutritional and therapeutic contexts.

The detailed chemical composition of nine wampee samples was determined using metabolomics analysis. Additionally, we sought to identify key compounds contributing to the distinct e-tongue response profiles and biological activities of wampee fruits. The results showed that most wampee samples were rich in flavonoids, amino acids and their derivatives, and alkaloids, which is consistent with findings from previous studies. Earlier analyses of wampee fruit composition have similarly reported flavonoids, organic acids, alkaloids, amino acids, coumarins, and terpenoids as the predominant chemical classes (Fan et al., 2020; Zhang, Wang, & Zhao, 2012). PCA revealed that the chemical compositions varied among different wampee fruits. W2, W4, W6, W7, W8, and W9 exhibited relatively similar metabolic profiles, while W1 displayed a markedly different composition. This compositional divergence may account for the significant differences in e-tongue responses observed between W1 and the other samples.

One of the most intriguing aspects of wampee's chemical composition is its high abundance of alkaloids. To our knowledge, few edible fruits contain alkaloids in such diversity and quantity as wampee. Notably, the alkaloids present in wampee fruits are predominantly carbazole alkaloids (Fan et al., 2021; Wang et al., 2024), a unique class of compounds with promising anticancer and neuroprotective properties (Iqbal Andrabi et al., 2024; Liu et al., 2019; Wang et al., 2024). In general, a high abundance of alkaloids in edible plants is often associated with potential toxicity. However, our *in vitro* and *in vivo* assessments demonstrated that wampee extracts rich in alkaloids exhibited minimal cytotoxicity or organ toxicity, supporting the safety of wampee as a functional food candidate. Compared with other abundant chemical classes in wampee, such as flavonoids, which can be obtained through various biosynthetic and extraction strategies (Shu et al., 2025), the biosynthesis and metabolic pathways of carbazole alkaloids remain underexplored. Further research into their biosynthesis is essential to fully harness their potential for therapeutic and functional applications.

The potential roles of different metabolites in determining the e-tongue responses and biological functions of wampee were explored using RFTA. The results indicated that only sourness and astringency were significantly influenced by specific metabolites such as vanilloloside, Ala-Leu-Leu, 4-caffeoylshikimic acid, and 2′-deoxycytidine. Among the compound classes, lignans and coumarins appeared to play a central role in shaping these two e-tongue response attributes. However, even for sourness and astringency, the IncNodePurity values suggested that no single compound had a dominant contribution to e-tongue responses, highlighting the complexity of e-tongue response formation in wampee. These findings differ from those of previous studies, such as Huang et al. (Huang et al., 2023), which identified 12 specific compounds as key contributors to wampee e-tongue response. The inconsistency may be attributed to variations in sample preparation, study design, and analytical methods, reflecting the broader lack of standardization in wampee research. Given these disparities, it is reasonable to advocate for future studies aimed at establishing standardized protocols for characterizing the sensory and functional properties of wampee. At least based on our findings, it can be inferred that the e-tongue response of wampee is more likely the result of complex interactions among multiple metabolites, rather than being driven by a few dominant compounds, as is the case in some other fruits (Song, 2018). Compared to e-tongue response attributes, the involvement of metabolites in the biological functions of wampee was more pronounced. In each RFTA analysis conducted for different biological activities, the IncNodePurity values were considerably higher, indicating a stronger influence of metabolite composition. Regarding antioxidant capacity, phenolic acids, flavonoids, and lipids emerged as the major contributing chemical classes, regardless of the detection method employed. In terms of anti-inflammatory activity, analysis of the top 30 metabolites in each assay suggested that alkaloids and flavonoids played central roles. Due to the complex chemical composition of wampee, identifying the exact compounds responsible for each biological function remains challenging. However, the comparative analysis of nine wampee fruits in the current study not only supports previous findings—such as those by Zhang et al. that highlighted the roles of phenolic acids, flavonoids, lipids, and alkaloid (Zhang et al., 2012), but also offers valuable insights for further elucidating the mechanisms underlying these bioactivities. Moreover, the findings provide a reference framework for future breeding programs and the development of functional products derived from wampee.

Collectively, the current study is the first to perform a comprehensive comparison of the metabolic composition between different samples of wampee, a tropical fruit with multiple biological activities and promising economic value. Moreover, we also attempted to link specific e-tongue response profiles and biological activities of wampee to specific compounds, and the data showed that the e-tongue responses of wampee formed *via* the complex interactions of multiple metabolites, and phenolic acids, flavonoids, lipids, and alkaloids were likely to be the key chemical classes contributing to the antioxidant and anti-inflammatory capabilities of wampee fruits. Based on the findings of the current study, future work should be performed by establishing standard nutrition criteria, selecting high-quality varieties, and breeding wampee varieties with improved tastes and biological functions, which will facilitate the development of the wampee-related industry.

## Author contribution

WHD and YYZ performed data curation, formal analysis, and wrote the draft. YL performed data curation and formal analysis. HZ performed data curation and formal analysis. KXL performed formal analysis. HZJ performed data curation. LDF performed data curation. HDC performed data curation. XYT performed data curation. ZYH performed data curation. NY performed data curation. SYF performed data curation. CS performed data curation, formal analysis, revised the draft. HBL performed formal analysis and revised the draft.

## CRediT authorship contribution statement

**Wen-hui Deng:** Writing – original draft, Formal analysis, Data curation. **Ying-ying Zheng:** Formal analysis, Data curation. **Yuan Li:** Formal analysis, Data curation. **Hong Zhang:** Investigation, Formal analysis. **Kai-xuan Lin:** Formal analysis, Data curation. **Han-Zhang Jiang:** Data curation. **Li-Dan Fu:** Data curation. **Hao-Dong Cui:** Data curation. **Xin-yue Tong:** Data curation. **Ziyang Hu:** Data curation. **Nan Yang:** Data curation. **Shi-yun Feng:** Data curation. **Chi Shu:** Writing – review & editing, Formal analysis, Data curation, Conceptualization. **Han-Bin Lin:** Writing – review & editing, Conceptualization.

## Fundings

This study was supported by research grants from the Key Research Project of Zhongshan Institute of Drug Discovery, 10.13039/501100002367Chinese Academy of Sciences (ZIDD202304 to H—B. L.), 10.13039/501100001809National Natural Science Foundation of China (82100391 to H—B. L.), Guangdong Provincial Pearl River Talent Program (211283781015 to H—B. L.), State key Laboratory of Drug Research of Shanghai Institute of Materia Medica Chinese Academy of Sciences (Grant Nos. SKLDR-2024-KF-08 to H—B. L.), High-level New R&D Institute (2019B090904008 to H—B. L.), High-level Innovative Research Institute (2021B0909050003 to H—B. L).

## Declaration of competing interest

The authors declare that they have no known competing financial interests or personal relationships that could have appeared to influence the work reported in this paper.

## Figures and Tables

**Figure:**
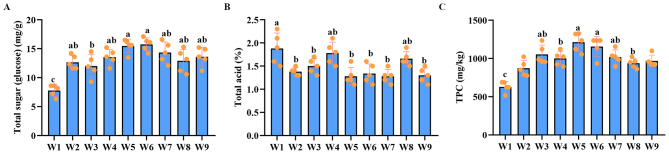
Supplementary Figure 1

**Figure:**
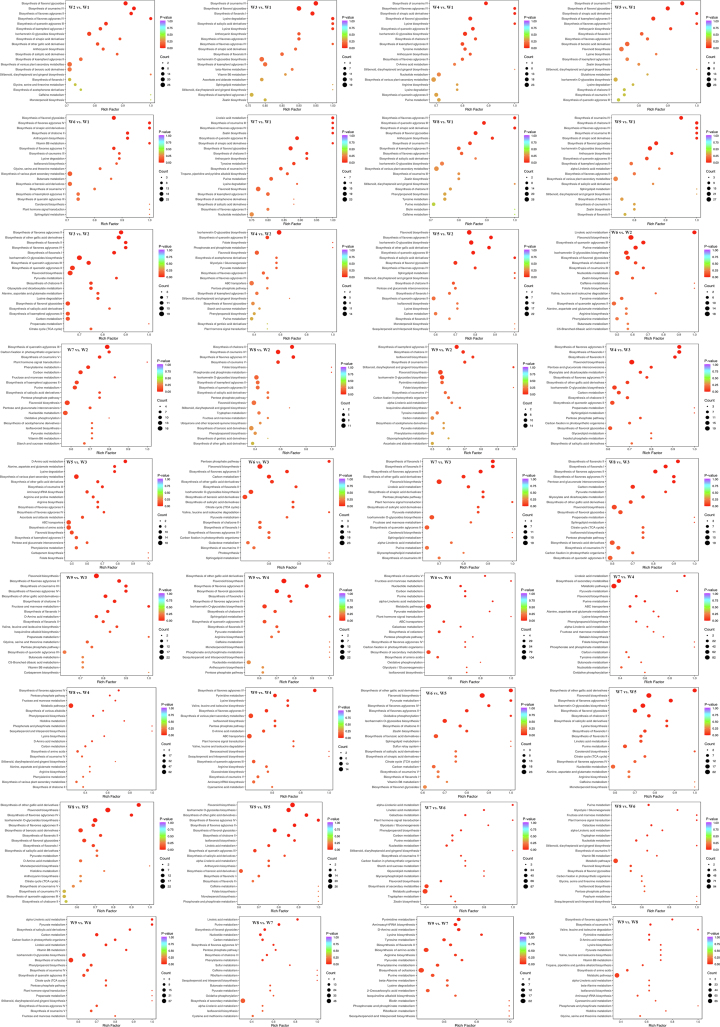
Supplementary Figure 2

## Data Availability

The data link is added in the MS.

## Appendix A Supplementary data

Supplementary material 1 Image 1

Supplementary material 2 Image 2

Supplementary material 3 Image 3
