# Supplementary material for: Phytochemical profile influences the e-tongue responses, antioxidant, anti-inflammation, and hypoglycemic effects of wampee fruits from different regions
Source: Food Chem X. 2025 Aug 5;29:102860. doi: 10.1016/j.fochx.2025.102860 (PMC12355570; doi:10.1016/j.fochx.2025.102860)
Supplement: Supplementary file 1 — Supplementary material 1 [file mmc1.docx]

**Figure S1 Total sugar, total acid, and total phenol content (TPC) of different wampee varieties. A**, total sugar represented by glucose of different wampee varieties. **B**, total acid of different wampee varieties. **C**, TPC of different wampee varieties.

**Figure S2 Differences regarding KEGG enrichment between each two wampee varieties.**
