# Supplementary material for: Phytochemical profile influences the e-tongue responses, antioxidant, anti-inflammation, and hypoglycemic effects of wampee fruits from different regions
Source: Food Chem X. 2025 Aug 5;29:102860. doi: 10.1016/j.fochx.2025.102860 (PMC12355570; doi:10.1016/j.fochx.2025.102860)
Supplement: Supplementary file 2 — Supplementary material 2 [file mmc2.docx]

**Table S1 Detailed information regarding the nine varieties of wampee fruits in the current study**

| **Label** | **Variety name** | **Average size (cm) and weight (g)** | **Location** |
| --- | --- | --- | --- |
| W1 | Baise shan wampee | 0.5 – 1.0 cm, 2 – 3 g | Baise City, Guangxi Province |
| W2 | Chouzuo seedless wampee | 2.0 – 4.0 cm, 7 – 8 g | Chouzuo City, Guangxi Province |
| W3 | Yongxing wild wampee | 1.5 – 3.0 cm, 7 – 9 g | Yongxing City, Hainan Province |
| W4 | Chonghua chicken-heart wampee | 3.0 – 5.0 cm, 9 – 14 g | Chonghua City, Guangxi Province |
| W5 | Yunan rock-candy wampee | 3.0 – 4.0 cm, 10 – 12 g | Yunan City, Guangdong Province |
| W6 | Yunan seedless wampee | 3.0 – 4.0 cm, 10 – 12 g | Yunan City, Guangdong Province |
| W7 | Lipu chicken-heart wampee | 2.8 – 3.3 cm, 9 – 12 g | Lipu City, Guangdong Province |
| W8 | Zhangzhou chicken-heart wampee | 2.5 – 3.0 cm, 10 – 12 g | Zhangzhou City, Guangdong Province |
| W9 | Beihai Black-diamond wampee | 3.0 – 5.0 cm, 12 – 15 g | Beihai City, Guangxi Province |
